# Supplementary material for: Modeling Risk Group 4 virus infection and antiviral treatment in microfluidic lung organ-on-chips in maximum containment laboratories
Source: bioRxiv. 2026 Aug 25:2026.08.24.745299. Preprint. [Version 1] doi: 10.64898/2026.08.24.745299 (PMC13585022; doi:10.64898/2026.08.24.745299)
Supplement: Supplementary file 1 [file NIHPP2026.08.24.745299V1-supplement-1.pdf]

bioRxiv preprint doi: https://doi.org/10.64898/2026.08.24.745299; this version posted August 25, 2026. The copyright holder for this preprint (which was not certified by peer review) is the author/funder. This article is a US Government work. It is not subject to copyright under 17 USC 105 and is also made available for use under a CC0 license.

Supplementary Figure 1

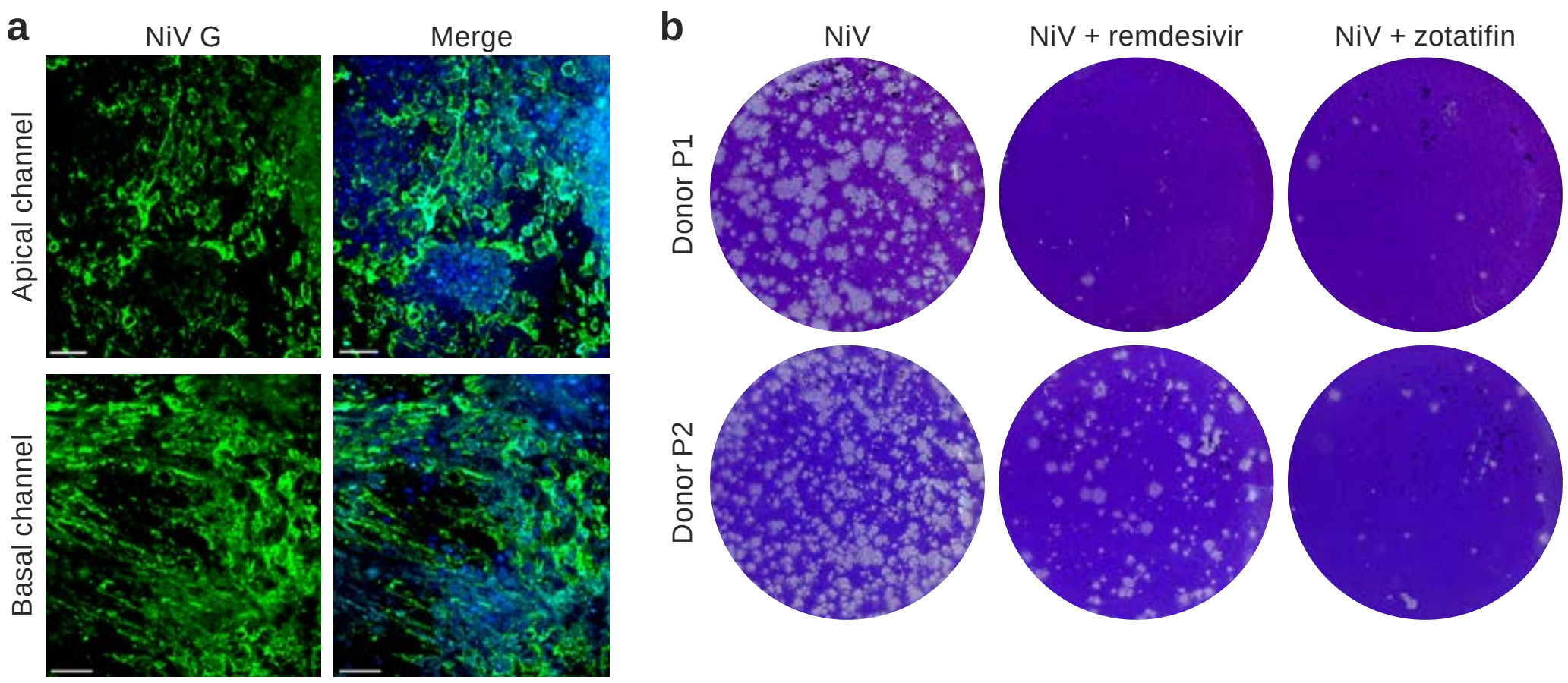

bioRxiv preprint doi: https://doi.org/10.64898/2026.08.24.745299; this version posted August 25, 2026. The copyright holder for this preprint (which was not certified by peer review) is the author/funder. This article is a US Government work. It is not subject to copyright under 17 USC 105 and is also made available for use under a CC0 license.

Supplementary Figure 2

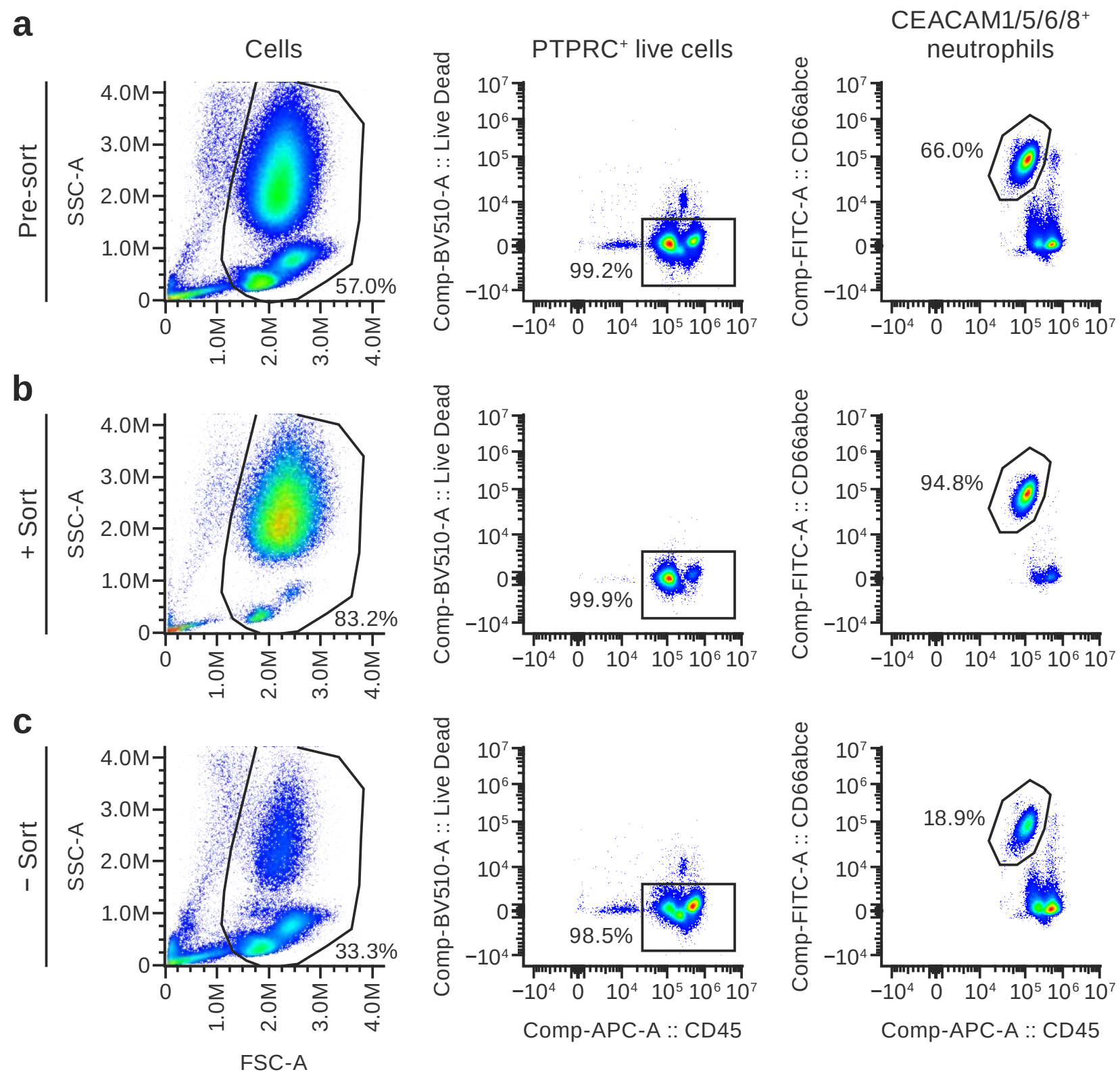

bioRxiv preprint doi: https://doi.org/10.64898/2026.08.24.745299; this version posted August 25, 2026. The copyright holder for this preprint (which was not certified by peer review) is the author/funder. This article is a US Government work. It is not subject to copyright under 17 USC 105 and is also made available for use under a CC0 license.

Supplementary Figure 3

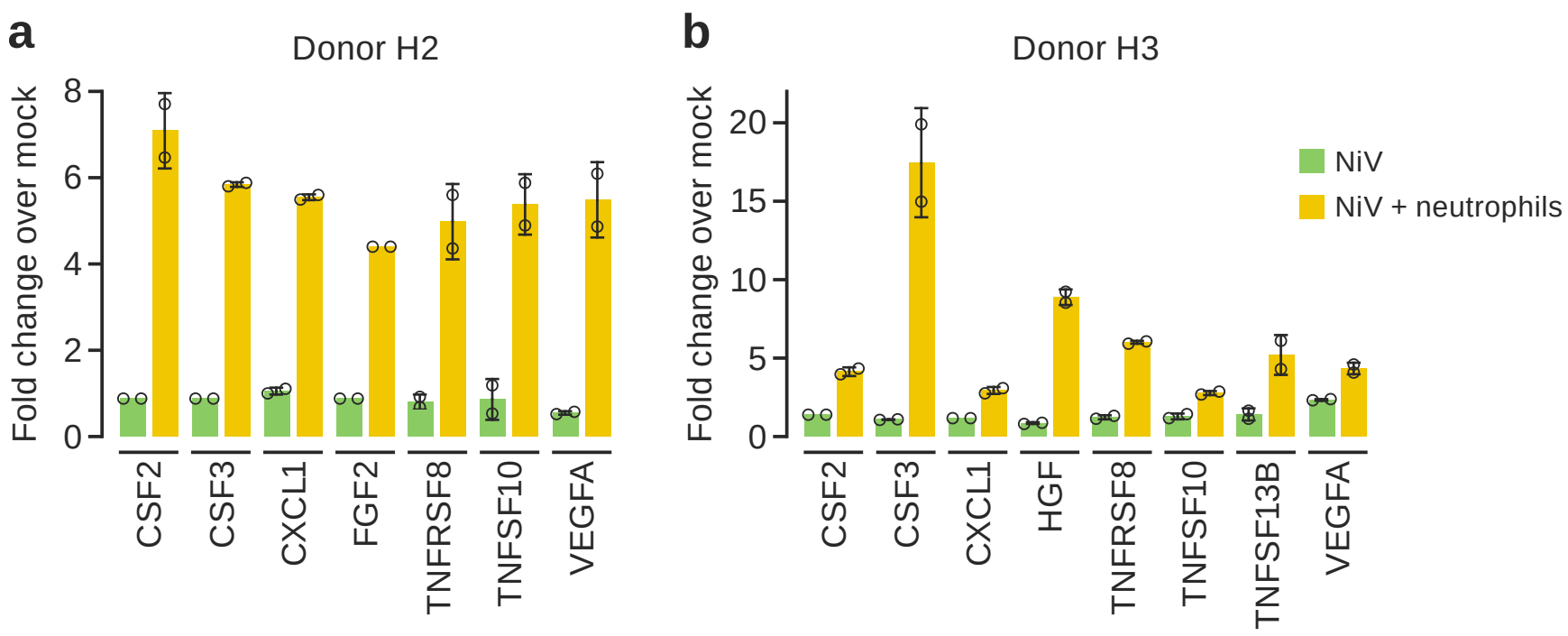

bioRxiv preprint doi: https://doi.org/10.64898/2026.08.24.745299; this version posted August 25, 2026. The copyright holder for this preprint (which was not certified by peer review) is the author/funder. This article is a US Government work. It is not subject to copyright under 17 USC 105 and is also made available for use under a CC0 license.

Supplementary Figure 4

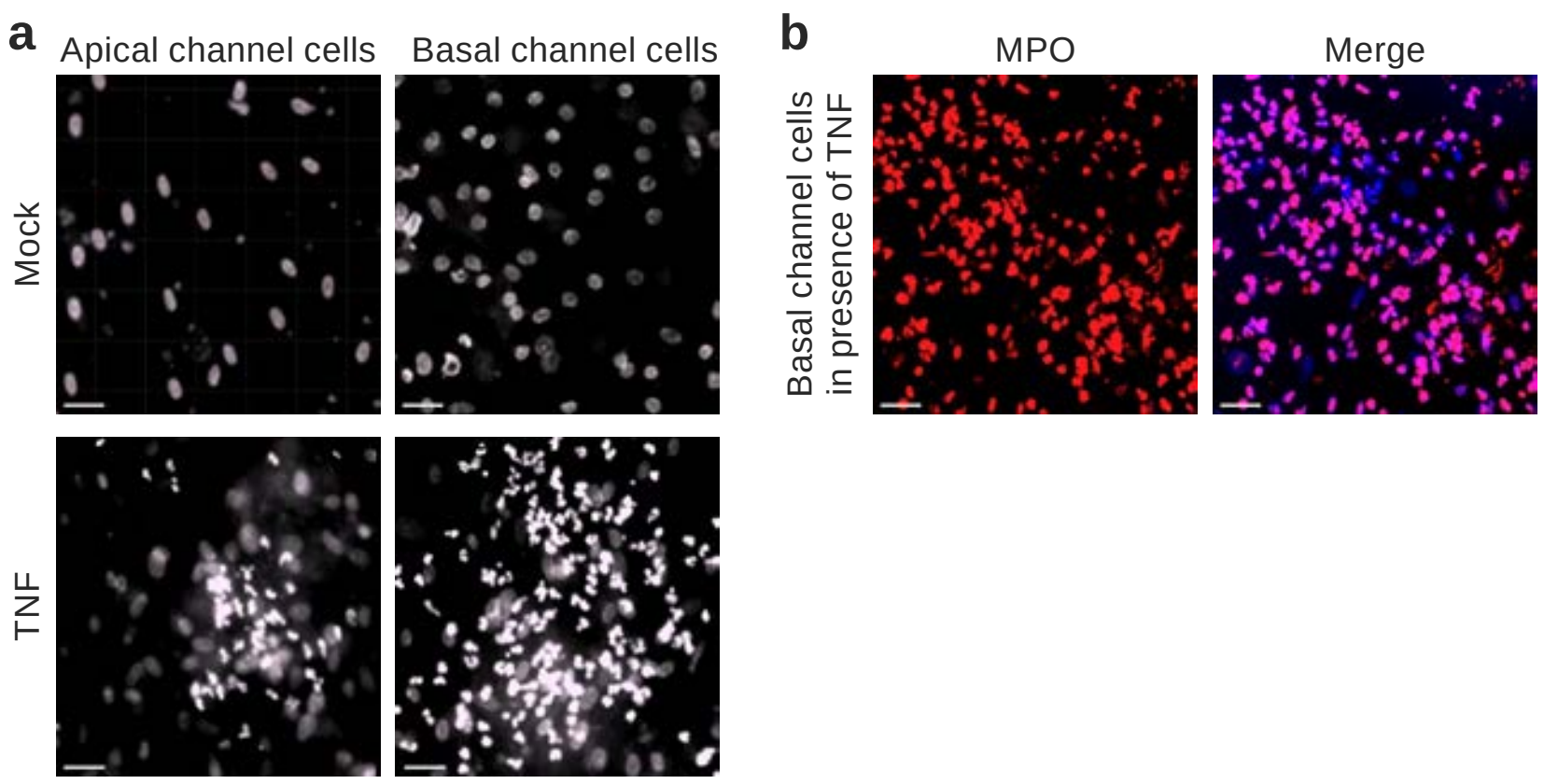

bioRxiv preprint doi: https://doi.org/10.64898/2026.08.24.745299; this version posted August 25, 2026. The copyright holder for this preprint (which was not certified by peer review) is the author/funder. This article is a US Government work. It is not subject to copyright under 17 USC 105 and is also made available for use under a CC0 license.

Supplementary Information

Modeling Risk Group 4 virus infection and antiviral treatment in microfluidic lung organ-on-chips in maximum containment laboratories

Sushma M. Bhosle[1], Julie P. Tran[1], Shuiqing Yu[1], Jillian Geiger[1], Arpita Das[1], Scott M. Anthony[1], Bapi Pahar[1], Rebecca Bernbaum-Cutler[1], Deja F. P. Rivera[1], Ian Crozier[2], Jiro Wada[1], Anya Crane[1], Gustavo Palacios[3,4], Nicole C. Kleinstreuer[5], Jens H. Kuhn[1,*] & Gabriella Worwa[1,*]

[1]Integrated Research Facility at Fort Detrick, National Institute of Allergy and Infectious Diseases, National Institutes of Health, Fort Detrick, Frederick, MD, USA
[2]Clinical Monitoring Research Program Directorate, Frederick National Laboratory for Cancer Research, Frederick, MD, USA
[3]Department of Microbiology, Icahn School of Medicine at Mount Sinai, New York, NY, USA
[4]Global Health Emerging Pathogens Institute, New York, NY, USA
[5] Division of Program Coordination, Planning, and Strategic Initiatives, National Institutes of Health, Research Triangle Park, NC, USA

bioRxiv preprint doi: https://doi.org/10.64898/2026.08.24.745299; this version posted August 25, 2026. The copyright holder for this preprint (which was not certified by peer review) is the author/funder. This article is a US Government work. It is not subject to copyright under 17 USC 105 and is also made available for use under a CC0 license.

Supplementary Figures

Supplementary Figure 1. Small-molecule antivirals reduce Nipah virus titers in lung organ-on-chips: Porcine alveolus lung organ-on-chips. a, Immunostaining of porcine pneumocytes (apical channel) and endothelial cells (basal channel) for Nipah virus (NiV) glycoprotein (G; green) and nuclei with Hoechst (blue). **b,** Representative NiV plaque assay enumerating virions in effluents collected from "untreated" and antiviral-"treated" porcine alveolus lung organ-on-chips.

bioRxiv preprint doi: https://doi.org/10.64898/2026.08.24.745299; this version posted August 25, 2026. The copyright holder for this preprint (which was not certified by peer review) is the author/funder. This article is a US Government work. It is not subject to copyright under 17 USC 105 and is also made available for use under a CC0 license.

Supplementary Figure 2. Nipah virus-induced neutrophil infiltration can be modelled in human small airway lung organ-on-chips. Freshly obtained human blood cells were stained to detect leukocyte common antigen, i.e., protein tyrosine phosphatase, receptor type, C (PTPRC) and CEA cell adhesion molecules 1/5/6/8 (CEACAM1/5/6/8) and sorted. The presorted (Pre-sort) (**a**), positively sorted (+ Sort) (**b**), and negatively sorted (- Sort) (**c**) cells were analyzed to detect neutrophil population percentages. The side-scatter cell population was gated and percentages of $PTPRC^{+}$+$CEACAM1/5/6/8^{+}$ neutrophils are shown in presorted, positively sorted, or negatively sorted groups.

bioRxiv preprint doi: https://doi.org/10.64898/2026.08.24.745299; this version posted August 25, 2026. The copyright holder for this preprint (which was not certified by peer review) is the author/funder. This article is a US Government work. It is not subject to copyright under 17 USC 105 and is also made available for use under a CC0 license.

Supplementary Figure 3. Nipah virus-induced neutrophil infiltration can be modelled in human small airway lung organ-on-chips: immune response in the presence of infiltrating neutrophils. Cytokine concentrations in basal small airway lung organ-on-chip (hsaOOC) effluents at 24 h after Nipah virus (NiV) exposure in absence or presence of neutrophils. CSF2, colony stimulating factor 2; CSF3, colony stimulating factor 3; CXCL1, C-X-C motif chemokine ligand 1; FGF2, fibroblast growth factor 2; HGF, hepatocyte growth factor; TNF, tumor necrosis factor; TNFRSF8, TNF receptor superfamily member 8; TNFSF10, TNF superfamily member 10; TNFSF13B, TNF superfamily member 13b; VEGFA, vascular endothelial growth factor A.

bioRxiv preprint doi: https://doi.org/10.64898/2026.08.24.745299; this version posted August 25, 2026. The copyright holder for this preprint (which was not certified by peer review) is the author/funder. This article is a US Government work. It is not subject to copyright under 17 USC 105 and is also made available for use under a CC0 license.

Supplementary Figure 4. Neutrophil infiltration in cells from apical and basal channels of human small airway lung organ-on-chips after tumor necrosis factor alpha (TNFA) treatment. (a) Small airway lung organ-on-chip (hsaOOC) treated with TNFA showed infiltrating neutrophils in cells from the apical and basal channels at 24 h. Bright field images were captured at 20X with EVOS7000 microscope; scale bar, 40 µm. b, Cells from basal channel stained for MPO1 (red). A merged image with nuclei (blue) is shown; scale bar, 40 µm. MPO, myeloperoxidase; TNF, tumor necrosis factor.

Supplementary Tables

Supplementary Table 1. Metadata for human donors of primary small airway epithelial and primary lung microvascular endothelial cells.

Human donor	Cell type	Lot number	Sex	Ethnicity	Age (yr)	Weight (kg)	Diabetes	Heart disease	Hypertension	Alcohol use	Smoking
H1	Human Small Airway Epithelial Cells (Lonza; Cat. #CC-2547)	21TL019234	Female	Caucasian	78	177	No	Unknown	Yes	No	No
H2	Human Small Airway Epithelial Cells (Lonza; Cat. #CC-2547)	22TL018267	Male	Caucasian	21	210	No	No	No	No	No
H3	HMVEC-L – Human Lung Microvascular Endothelial Cells (Lonza; Cat. #CC-2527)	21TL169356	Female	Caucasian	2	34	No	No	No	No	No

Supplementary Table 2. Metadata for porcine donors of primary alveolar epithelial and primary lung microvascular endothelial cells.

Porcine donor	Cell type	Lot number
P1	Porcine Primary Alveolar Epithelial Cells (Cell Biologics; Cat. #P-6053)	061913NAB.JS
P2	Porcine Primary Alveolar Epithelial Cells (Cell Biologics; Cat. #P-6053)	M051623W12
P3	Porcine Primary Lung Microvascular Endothelial Cells (Cell Biologics; Cat. #P-6011)	012616.JS

Supplementary Table 3. Antibodies used in immunofluorescence assays.

Target Antigen	Antibody	Manufacturer/Provider	Cat. #	Organ-on-chip	Channel	Biosafety level
**Primary antibodies**						
Acetylated tubulin	Acetyl-α [acetylated] Tubulin (Lys40) (D20G3) XP® Rabbit mAb	Thermo Fisher Scientific	PA5-47488	hsaOOC	Apical	2
CDH1	Anti-E Cadherin antibody - Intercellular Junction Marker	Abcam	Ab15148	hsaOOC	Apical	2
CDH5	CD144 (VE-cadherin) Monoclonal Antibody (16B1), eBioscience	Thermo Fisher Scientific	14-1449-82	hsaOOC, paOOC	Basal	2
EFNB2	Human/Mouse/Rat Ephrin-B2 polyclonal antibody	R&D Systems	AF496	hsaOOC, paOOC	Apical and basal	2
MKI67	Ki-67 Monoclonal Antibody (SolA15), FITC, eBioscience	Thermo Fisher Scientific	11-5698-82	hsaOOC, paOOC	Apical	2
MPO	Rabbit Myeloperoxidase Polyclonal Antibody	Thermo Fisher Scientific	PA516672	hsaOOC	Apical and basal channels (in presence of TNF)	4
NiV G	Nipah virus glycoprotein G antibody, customized	Christopher Broder, Uniformed Health Services University	PA8905	hsaOOC	Apical and basal	4
PECAM1	CD31 Polyclonal Antibody	Thermo Fisher Scientific	PA5-32321	hsaOOC, paOOC	Basal	2
TJP1	ZO-1 Monoclonal Antibody (ZO1-1A12), Alexa Fluor 594	Thermo Fisher Scientific	339194	hsaOOC, paOOC	Apical	2
Nucleic acid	Hoechst 33342, trihydrochloride trihydrate	Thermo Fisher Scientific	H3570	hsaOOC, paOOC	Apical and basal	2
**Secondary antibodies**						

/	Donkey anti-Goat IgG (H+L) Cross-Adsorbed Secondary Antibody, Alexa Fluor 647	Thermo Fisher Scientific	A-21447	hsaOOC	Apical	2
/	Donkey anti-Mouse IgG (H+L) Highly Cross-Adsorbed Secondary Antibody, Alexa Fluor Plus 594	Thermo Fisher Scientific	A-32744	hsaOOC	Apical	2
/	Donkey anti-Rabbit IgG (H+L) Highly Cross-Adsorbed Secondary Antibody, Alexa Fluor 488	Thermo Fisher Scientific	A-21206	hsaOOC	Apical	2, 4
/	Donkey anti-Rat IgG (H+L) Highly Cross-Adsorbed Secondary Antibody, Alexa Fluor 594	Thermo Fisher Scientific	A-21209	hsaOOC	Apical	2

CDH1, cadherin 1; CDH5, cadherin 5; EFNB2, ephrin B2; hsaOOC, human small airway lung organ-on-chip; MKI67, marker of proliferation KI-67; MPO, myeloperoxidase; NiV G, Nipah virus glycoprotein; paOOC, porcine alveolus lung organ-on-chip; PECAM1, platelet and endothelial cell adhesion molecule 1; pro-SFTPB, pro-surfactant protein B; TNF, tumor necrosis factor; TJP1, tight junction protein 1; VWF, von Willebrand factor.
